# Supplementary material for: Alcohol consumption increases non-adherence to ART among people living with HIV enrolled to the community-based care model in rural northern Uganda
Source: PLoS One. 2020 Nov 24;15(11):e0242801. doi: 10.1371/journal.pone.0242801 (PMC7685449; doi:10.1371/journal.pone.0242801)
Supplement: Attachment — Submitted filename: PLOS Review Alcohol consumption increases non.docx [file pone.0242801.s002.docx]

Alcohol consumption increases non-adherence to ART among people living with HIV enrolled to the community-based care model in rural northern Uganda

Line	Comment

Abstract

46	Rather wide 95%CI, an effect of sample size?

47-50	What was the rationale of choosing the other factors?

Introduction

61-63	Is this the population of the region? Latest population of SSA is 1.1 billion?

70-73	Correct grammar here!

90-92	Address the grammar not reading well.

Study population: eligibility criteria, sample size, and sampling

125-131	What were the assumptions made on the proportion of the non-adherent, the odds ratio’s 95% CI is wide, suggesting possibility of a small sample size? Please outline the assumed prevalence!

136-138	It’s unclear how the sampling was done, was it simple random sampling or systematic random sampling? Please be clear

Measurements

144-154	As this is a cross sectional study, it is not appropriate just to assume a variable to be predictor or outcome, is there any particular reason to take these to be predictor and outcome variables?

Data collection and quality control measures or outcomes

160-162	Address grammar here?

Ethical Issues

186-188	Address the grammar here.

Table 2.

212	Is this breakdown based on the 25 who were non-adherent or the whole sample, eg forgetfulness 45 vs 25?

Table 4.

257-258	Please take care of grammar. None Acholi or non-Acholi

Discussion

264-265	Dichotomizing ART adherence was always going to be a problem, viral loads were better in assessing adherence.

270-274	Is this a finding from the study or from elsewhere, ref if possible.

276-279	In line with my comment above, please explain where we are getting this from. The frequencies are not related to the 25 found in the study?

287-293	Discussion is based on the study findings, this is not from this study. I would omit it in write-up!

313-320	I would suggest you limit extensive discussion on the main research question, ART and alcohol consumption!

322-326	This is this redundant, unrelated to the study questions?

335-337	Standard definition is not the best though, viral suppression is probably the best, this is a major limitation!

338-340	Another limitation, alcohol consumption was dichotomized, why not use a tool, eg the AUDIT?
